# A scoping review of current practices on community engagement in rural East Africa: Recommendations for snakebite envenoming

**DOI:** 10.1016/j.toxcx.2021.100073

**Published:** 2021-07-16

**Authors:** Bethany Moos, David Williams, Isabelle Bolon, Denise Mupfasoni, Bernadette Abela-Ridder, Rafael Ruiz de Castaneda

**Affiliations:** a Hedena Health, 207 London Road, Headington, Oxford, UK; b Department of Control of Neglected Tropical Diseases, World Health Organization, Avenue Appia 20, CH-1211, Geneva 27, Switzerland; c Institute of Global Health, Department of Community Health and Medicine, Faculty of Medicine, University of Geneva, Campus Biotech, Chemin des Mines 9, CH-1202, Geneva, Switzerland; d Division of Tropical and Humanitarian Medicine & Institute of Global Health, Department of Community Health and Medicine, Faculty of Medicine, University of Geneva, Geneva, Switzerland

**Keywords:** Snakebite envenoming, Neglected tropical diseases, Community engagement, Public health, One health, Communication

## Abstract

Community empowerment and engagement is one of the four strategic aims highlighted in the WHO strategy to prevent and control snakebite envenoming. Inappropriate health-seeking behaviours contribute to adverse outcomes, and community engagement is key in driving behavioural change. WHO has highlighted East Africa as a geographical area of concern for snakebite envenoming. The overall aim of the project is to develop a community engagement toolkit for snakebite envenoming and other NTDs. The objective of this scoping review was to identify current practices in recent community engagement in rural East Africa; the applicability of these results to snakebite envenoming are discussed. *PubMed, Web of Science*, *PsycINFO* and *Google Scholar* were searched from 1 January 2017 to 3 September 2020. Search terms were used to identify publications which related to rural communities and health or disease, for both humans and animals. After reviewing the full papers for all geographical areas, 112 publications were included, 30 of which were conducted in East Africa. Papers included nine different countries and covered a broad range of health topics; notably, water, sanitation and hygiene, nutrition, and maternal and child health. Only one publication considered animal health. The most common form of engagement was in the context of a group meeting, lecture, presentation, discussion or question and answer session (63.3%). A variety of locations within the community were used to engage with people, the most common being an individual's household (23.3%). Communication factors was the key influencer for engagement, both positively and negatively. Key barriers to engagement include local languages and health beliefs, literacy levels, mobile phone ownership and the level of mobile Internet coverage, burden of agricultural work and weather conditions. This study provides an extensive overview of recent public health community engagement in East Africa, which will serve as a useful resource for any group seeking to plan an intervention in remote and rural areas in East Africa. Furthermore, it serves as a guide to help tailor community engagement to snakebite envenoming.

## Highlights

• This review includes 30 publications from nine East African countries; only one considered animal health.
• The most common method of engaging with communities was in a group setting, followed by household visits.
• Areas highlighted for cross-sectoral collaboration include water, sanitation and hygiene, nutrition and maternal and child health.
• Communication was the main factor that influenced engagement, positively or negatively.
• Other key barriers to engagement include poverty, the climate, burden of agricultural work and traditional health beliefs.

## 1 Introduction

There remains a lack of consensus over a definition of community engagement (CE); however, the World Health Organization (WHO) defines it as “a process of developing relationships that enable stakeholders to work together to address health-related issues and promote well-being to achieve positive health impact and outcomes” (WHO, 2017). The United States Centers for Disease Control and Prevention (CDC), as cited by the Clinical Translational Science Awards Consortium (2011) highlights CE as a “powerful vehicle for bringing about environmental and behavioural changes that will improve the health of the community and its members” (Clinical Translational Science Awards Consortium, 2011). Indeed, a change in knowledges, attitudes and perceptions is not enough to drive a change in behaviour, especially when it is deeply intertwined in the very fabric of culture itself. This can be seen as victims of snakebite continue to seek care from traditional healers, rather than from a healthcare facility, despite better availability of snake antivenom (Schioldann et al., 2018).

Snakebite envenoming (SBE) is a disease affecting the rural poor that is responsible for up to 138,000 deaths worldwide every year, with many more left suffering with permanent disability or disfigurement (Seventy-first World Health Assembly, 2018). Globally the three areas with the highest number of envenomings include South Asia, South-East Asia, and East Sub-Saharan Africa (Kasturiratne et al., 2008). Chippaux (2011) estimates up to 14,600 amputations occur per year secondary to SBE in Sub-Saharan Africa; East Africa is estimated to have 5177 deaths, 6127 amputations and 22,941 cases of post-traumatic stress disorder secondary to SBE (Halilu et al., 2019). There are several countries in Sub-Saharan Africa where the data on the number of envenomings or deaths remain limited or absent. Data from Halilu et al. (2019) provides the mean number of cases of SBE, which ranges from 61 cases in Djibouti, up to 35,633 cases in Ethiopia. The estimated number of cases of SBE for the remaining East African countries included in the study are: Mauritius 763, Somalia 3,315, Burundi 3,870, Zambia 3,940, Zimbabwe 4,066, Rwanda 4,171, Malawi 5,855, Mozambique 6,996, United Republic of Tanzania 13,504, Uganda 13,801 and Kenya 15,411. Rural areas in Sub-Saharan Africa are disproportionately affected, with an incidence of SBE approximately 10 times higher compared to urban areas (Chippaux, 2011). For this study, the United Nations classification was used to classify countries into geographical regions; East Africa comprises British Indian Ocean Territory, Burundi, Comoros, Dijbouti, Eritrea, Ethiopia, French Southern Territories, Kenya, Madagascar, Malawi, Mauritius, Mayotte, Mozambique, Réunion, Rwanda, Seychelles, Somalia, South Sudan, Uganda, United Republic of Tanzania, Zambia and Zimbabwe (The United Nations Statistics Division, 1999).

The health beliefs and behaviours surrounding snakebite vary enormously, even within a country. The use of traditional medicine or techniques such as tourniquet use, tattooing, burning, cutting or placing a stone or herbal medicine over the bite site remain commonplace. Those bitten by snakes will often seek care from a traditional healer rather than presenting to a healthcare facility. Indeed, in Sub-Saharan Africa, at least 75% of victims opted for traditional medicine as first-line treatment (Chippaux, 2011). There may be multiple reasons for these actions, such as a perceived or actual lack of adequate medical facilities, an inability to afford treatment, issues with transport to a healthcare facility or a mistrust of services. Furthermore, traditional healers often hold a position of influence within communities as respected and trusted individuals. The use of traditional treatments, in addition to the infrequent use of the correct first-aid management of snakebite, contributes to the current high burden of morbidity and mortality from SBE. Furthermore, an understanding of, and engagement with, the preventive measures to avoid a snakebite is crucial. An educated community is an empowered community, and it is well-recognized that CE is a fundamental component in reducing the impact and negative outcomes of SBE (Kadam et al., 2021).

In May 2019, the WHO released the snakebite roadmap, outlining its strategy to halve death and disability from snakebite by 2030 (Seventy-first World Health Assembly, 2018; World Health Organization, 2019). Community empowerment and engagement, specifically through education and training, is one of four strategic aims highlighted in the roadmap. Like a number of other neglected tropical diseases (NTDs), this disease is both preventable and treatable, and effective engagement with affected communities may be the most cost-effective approach to improving outcomes. It is essential to consider the local knowledge, attitudes and health beliefs on SBE at the community level in order to ensure that a CE intervention is targeted to the need and is culturally appropriate. CE should focus on, but not be limited to, the prevention of SBE and appropriate first-aid management. Community services should be in place to support victims, who are left not only with long-term physical disability but also with psychological sequelae such as depression and post-traumatic stress disorder (Bhaumik et al., 2020).

There are many shared gains to be made from a cross-cutting approach to the NTDs and consideration to other issues that affect rural and impoverished communities by collaborating with other groups (WHO, 2020). A move away from siloed approaches will facilitate a recovery from the devastating blow of the COVID-19 pandemic, as we work towards the targets set out by the Sustainable Development Goals and, ultimately, to achieve universal health coverage (UHC). It is not possible to develop a ‘one size fits all approach’ for CE; a customized approach is not desirable, but rather is essential to the success of such programmes. For NTDs, which largely affect the rural poor, CE underpins the entire process of building sustainable NTD capacity (Ortu and Williams, 2017). Specifically, for SBE, a critical action outlined in the NTD road map is to “build awareness in communities on best practices in prevention and seeking treatment for snakebite envenoming” (WHO, 2020). Furthermore, the WHO snakebite roadmap emphasizes the impact of SBE on animal health and the importance of a transdisciplinary alliance between veterinary and human medicine for a ‘One Health’ approach to prevention and control of SBE (WHO, 2019).

The overall aim of the project is to develop a CE toolkit for SBE and other NTDs, with a framework of the key components that will ensure that interventions are strategically and logically planned, implemented and evaluated. This toolkit will be adaptable to any country and community and will contribute to the WHO's aim of reducing morbidity and mortality secondary to SBE across the globe. To guide the development of this toolkit, this scoping review was conducted to explore current practices on CE with people living in remote and rural communities, such as those affected by NTDs. The objective of the scoping review is to identify current practice in recent CE that focuses on rural communities in East Africa, an area of high morbidity and mortality highlighted by the WHO as a geographical area of concern (WHO, 2019). We provide an overview of the interventions and those who deliver them, in addition to factors that both enable and hinder CE on human and animal health, in keeping with the WHO strategy for SBE prevention and control (Williams et al., 2019). This scoping review discusses the applicability of these results to SBE and how best practice might be achieved for those planning a CE intervention on SBE.

## 2 Materials and methods

### 2.1 Review protocol

The scoping review protocol was designed based on guidance for scoping reviews from the Joanna Briggs Institute (Aromataris and Munn, 2020), Arksey and O'Malley (2005) and Levac, Colquhoun & O'Brien (2010). The Preferred Reporting Items for Systematic reviews and Meta-Analyses extension for Scoping Reviews (PRISMA-ScR) checklist was also used in both the protocol design and the reporting of the study (see Appendix A) (Tricco et al., 2018).

### 2.2 Search strategy

*PubMed*, *Web of Science*, *PsycINFO* and *Google Scholar* were searched to find relevant publications. The search was conducted on 3 September 2020, including studies published from 1 January 2017 to the search date. There was no limitation on publication language. We used *Google Scholar* and *PsycINFO* to capture ‘grey literature’. A selection of keywords were used which related to rural communities, health or disease, and knowledge, attitude, behaviour, morbidity or mortality. To search *PubMed*, the following search string was used: Title/Abstract (Rural OR Communit* OR Agricultural OR Agrarian OR Farming OR Farmer) AND (disease OR health OR NTD OR NZD OR Zoono* OR “Snake bite” OR Snakebite) AND (Knowledge* OR Attitude* OR Behaviour* OR Behavior* OR Practice* OR Awareness OR Morbidity OR Mortality OR Death OR Fatality) AND Title (Communication* OR “Capacity building” OR Educat* OR Campaign OR Program* OR Toolkit OR Toolbox). The full search strings used for each database can be seen in Appendix B.

## 3 Inclusion and exclusion criteria

Research that conducted an intervention and evaluated a change in behaviour or practice, or an impact on either human or animal health, was included; both qualitative and quantitative research were considered. Reviews, meta-analyses and descriptive studies were excluded. Countries in East Africa, as per the United Nations classification, were included. The focus was on rural populations, hence publications were excluded if there was a mixture of urban and rural communities, unless a subgroup analysis was available for the rural population. The ‘community’ was defined as those living in a rural area who were at risk of SBE. Whilst some CHWs live and work in communities at risk of SBE, this was not always clear from the publications. All healthcare workers, in either the community or hospital setting, were excluded given their professional role, as they were often key individuals who delivered CE to the community. Where healthcare workers received education or training, this was considered as training the trainer.

All geographical locations across the globe were considered; however, publications from high-income countries (The World Bank,) were included only if the health topic addressed was an NTD. The overall ongoing project focuses on all countries across the globe; however, this publication presents the results for East Africa only, given the high burden of disease and level of concern highlighted in WHO's snakebite roadmap (World Health Organization, 2019). Initially, the search was conducted to capture publications from the preceding five years. However, the timeframe was further restricted to 3.5 years. Whilst the initial search included publications that measured a change in knowledge, attitude or practice, the decision was made to exclude these after the initial screen given that these do not necessarily translate into behaviour change or have an impact on health. Publications with no clear relevance to the topic were excluded.

## 4 Screening process

The list of publications was imported from all databases into EndNote X9 software. Duplicates were then removed. Initially, a pilot of 200 publications was done independently by three reviewers (BM, RRdC and IB) to compare consistency and to support the methodology of using a single reviewer (BM). After review of the first 100 publications, there was agreement on 63% of the decisions to include or exclude. The areas of inconsistency were discussed until there was a consensus. On review of the next 100 publications, there was agreement on 91% of papers, with only 9% that required discussion. Thus, a single expert reviewer (BM) independently reviewed the publications by title and abstract, a methodology also supported by Waffenschmidt (2019). Where there were uncertainties, publications were reviewed by two additional reviewers (RRdC and IB). Included publications were read in full and further papers were excluded.

## 5 Charting data

Data was extracted into a data charting form using Excel. Data charted included key study identifiers, the study population, details about the CE intervention (health topic, objective of study, target group and sample size), how it was delivered (forms of communication and engagement used, group size, total number of sessions delivered and their length, data about the person delivering the CE intervention, location of the CE, languages used and information about what either facilitated or hindered CE) and the indicators measured (a change in behaviour or practice, or an impact on health).

## 6 Results

### 6.1 Characteristics of publications

**Fig. 1:**
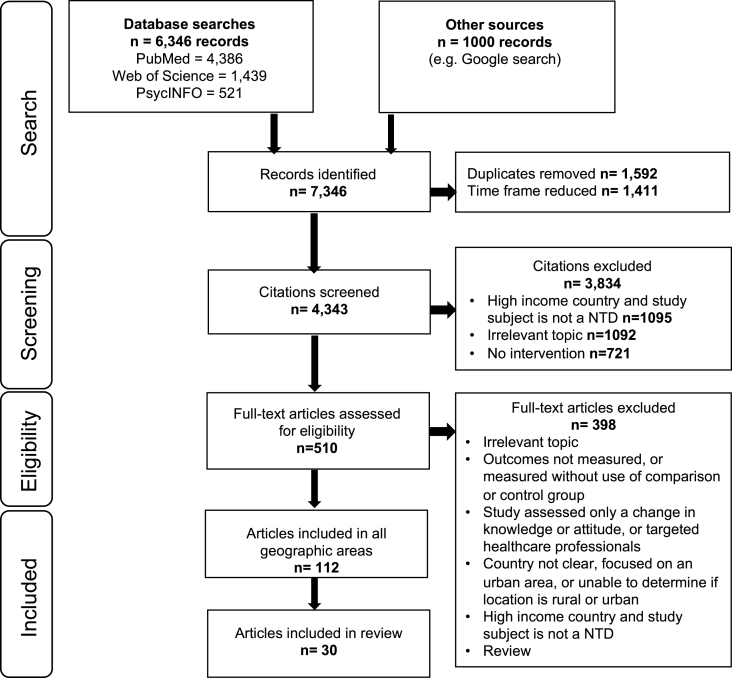
PRISMA flowchart of the selection process of publications to include in the scoping review.

The search retrieved 7346 publications; after duplicates were removed and the timeframe reduced, 4343 publications remained. Following the initial screen by title and abstract, 510 publications required review by full paper. There were 112 included publications after reviewing the full papers for all geographical areas, 30 of which were conducted in East Africa and are therefore included in this review (Fig. 1). All publications were in English, covering nine different countries; the largest number of publications were from the United Republic of Tanzania (n = 7) (Table 1.). No publications for the other East African countries met the inclusion criteria. Publications covered a broad range of health topics (Table 2.); specifically, within the category of NTDs, porcine cysticercosis (6.7%), soil-transmitted helminthiases (6.7%), schistosomiasis (3.3%), lymphatic filariasis (3.3%) and trachoma (3.3%) were featured. The majority of papers (96.7%) focused solely on human health; however, one publication considered both human and animal health (Chilundo et al., 2018).

**Table 1:** Characteristics of included publications.

Category		Count
Language	English	30

Year of publication	2020	7
	2019	8
	2018	10
	2017	5

Country	United Republic of Tanzania	7
	Ethiopia	5
	Kenya	5
	Malawi	5
	Uganda	4
	Mozambique	2
	Rwanda	1
	Zambia	1

**Table 2:** Health topic of included publications.

Category	Count (%)
Water, sanitation and hygiene	10 (33.3)
Nutrition	8 (26.7)
Child health	7 (23.3)
Maternal health	7 (23.3)
Neglected tropical diseases	3 (10.7)
Cervical cancer	3 (10.7)
Health finance	2 (6.7)
Food security	2 (6.7)
Helminth control	2 (6.7)
Violent behaviour	2 (6.7)
Diabetes	1 (3.3)
Intestinal infections	1 (3.3)
Mental health	1 (3.3)
Respiratory infections	1 (3.3)

### 6.2 Target population

The most common sociodemographic groups targeted were mothers or caregivers (33.3%) and pregnant women (13.3%). Four publications target children, one of which specifies that these are schoolchildren. Given that this scoping review focuses on rural areas, it is also interesting to note that 10% of publications specifically target farmers.

### 6.3 Communication

The language used for the CE was not documented in 50% of publications. When documented, between one and three different languages were used to communicate. Publications tended to use a variety of forms to communicate and engage with rural communities; public (66.7%), group (46.7%), mass (43.3%) and interpersonal (43.3%). Traditionally, there are five forms of communication; intrapersonal, interpersonal, group, public, and mass (LibreTexts). Intrapersonal communication represents an internal dialogue and hence was not a relevant category for this study. Interpersonal communication comprises a “two-way, nonmediated message exchange between a very small number” of people, whereas conversely mass communication was defined as “one-way, technologically mediated messages, delivered to large audiences” (O'Sullivan and Carr, 2017). Group communication entails an interactive participatory process between at least three people, whereas if within a group of people one person is delivering information to an audience, this is defined as “public” (LibreTexts). A wide range of different methods were used to mobilize communities or engage with them as part of the study intervention (Table 3). Large group events were most commonly used to communicate (63.3%), followed by communication on an individual level at a visit to the household (30%).

**Table 3:** Methods of community engagement.

Community engagement methods	Count (%)
Group meetings, lectures, presentations, discussion, question and answer sessions	19 (63.3)
Household visit	9 (30)
Community events, activities or announcements	8 (26.7)
Demonstration	7 (23.3)
Cooking session or show	5 (16.7)
Radio broadcast	5 (16.7)
Mobile phone (call or SMS or App)	4 (13.3)
Poster	4 (13.3)
Leaflet	3 (9.7)
Billboard	2 (6.7)
Games	2 (6.7)
Individual verbal discussion	2 (6.7)
Psychotherapy/counselling (group)	2 (6.7)
Roleplay or drama	2 (6.7)
Song	2 (6.7)
Workshop	2 (6.7)
Calendar displaying a health message	1 (3.3)
Psychotherapy/counselling (individual)	1 (3.3)
T-shirt displaying a health message	1 (3.3)

Most publications were solely CE interventions, but one third (33.3%) included other interventions, such as a medical treatment. Where education was given in a group setting, there was great variability in the number of people within the group (ranging from 10 to 200), in the number of sessions delivered (ranging from 1 to 24) and in the duration of the session (ranging from 20 minutes to a full day).

### 6.4 Location of community engagement intervention

A variety of different locations within the community were used to engage with people; the most common site documented was the more private location of an individual's household (23.3%), in comparison to the other more public locations within the community (Table 4).

**Table 4:** Location of intervention.

Location of intervention	Count (%)
No location documented	15 (50.0)
Household	7 (23.3)
Clinic	4 (13.3)
School	3 (10.0)
Public place in community	3 (10.0)
Farm	2 (6.7)
Church	1 (3.3)
Distribution site	1 (3.3)
Market area	1 (3.3)
Tents	1 (3.3)
Not applicable	1 (3.3)

### 6.5 Those delivering the engagement

There was a diverse number of different groups delivering the CE intervention; from health workers in the community (such as nurses or community health workers), to external agents (such as the study team or nongovernmental organization staff), to individuals within the communities (such as students and teachers). Community health workers, community health volunteers, nurses and the study team were most commonly documented to be delivering the CE. Approximately half of people in these roles were documented to have had a form of training for their role, although in 43.3% of cases this information was not recorded. Individuals received a salary or an incentive in 3.3% and 6.7% of studies respectively.

### 6.6 Factors that affected engagement with the community

The 30 publications highlighted a number of different factors that facilitated or led to positive engagement, and similarly that either hindered or led to less effective engagement with the community (Table 5) (see the full list in Appendix C). Influencing factors were grouped under the following headings: communication factors, financial factors, intervention factors, cultural factors, delivery of the intervention, people delivering the intervention, physical location of the intervention, the wider community, environmental factors, technological factors and ‘other’. Communication factors were the key influencer for engagement, both positive and negative. Use of other literate individuals, pictures, visual aids, models and even preparation of recipes aided communicating with those who were illiterate, which is a common barrier to engagement in remote and rural settings. Interpreters were required for local languages, and this proved problematic at points requiring bi or tri-lingual interpreters; one publication highlights that lack of certain words existing in the local language, such as cancer, in either the verbal or written form (Lidofsky et al., 2019). Where interventions were tailored to a community or setting, a positive impact was identified. However, two publications observed the detrimental impact of traditional health beliefs in affecting a change in health-seeking behaviour (Chiutsi-Phiri et al., 2017; Ekirapa-Kiracho et al., 2017).

**Table 5:** Factors that positively or negatively impacted on community engagement.

Category	Number of factors that facilitated or led to effective engagement	Number of factors that hindered or led to ineffective engagement
Communication factors	10	8
Financial factors	3	8
Intervention factors	7	2
Cultural factors	1	6
Delivery of intervention	3	3
The wider community	3	3
People delivering the intervention	5	2
Environmental factors	0	5
Technological factors	0	2
Physical location of intervention	1	0
Other	4	6

The use of a participatory approach to engagement by involving the community in the decision-making and the use of a package of interventions rather than a single area being targeted were highlighted as factors that facilitated engagement. One study developed a model of sustainable village governance to support the existing programme to control NTDs; the model placed ‘the village at the centre of the reform process’ and this highlighted relevant issues that related to the power and control between those implementing the program, the social services committee, the community and other actors (Madon et al., 2018). A nutrition education program that included practical cooking sessions attributed the successful behaviour change to the use of participatory approaches used in the education sessions (Chiutsi-Phiri et al., 2017). A Maternal and Neonatal Implementation for Equitable Systems (MANIFEST) project used a participatory approach for implementation which involved giving the local stakeholders responsibility for selecting the approaches used; they were able to make changes as they felt appropriate (Ekirapa-Kiracho et al., 2017). This was reported to promote ‘social approval and ownership of the project, which reduces resistance to the uptake of interventions’ (Ekirapa-Kiracho et al., 2017). The background of the study team, the support and supervision that they received and positive personal characteristics were of note. The social network of study participants was also observed in some cases to have a substantial positive influence on adopting sustainable behaviour change. Conversely, where support from family or friends was lacking, this negatively impacted on participation. Two studies described the association of stigma with the condition under study, impacting both engagement with and access to services (Mutamba et al., 2018; Settergren et al., 2018). Some diseases may be stigmatizing to all those affected or particular sociodemographic groups, such as women. An in-depth understanding of the disease itself, in addition to the complex social context, is essential in order to successfully engage with those affected by stigma. Particular attention should be paid to those who are engaging with communities, to ensure they are trusted and respected individuals who will be able to reach these hard to access groups. If women are experiencing stigma, then CE is more likely to be successful if those conducting CE are also female. Furthermore, a more private location may be appropriate if individuals are being targeted, rather than a public arena.

Financial issues for the study team were problematic at times, in addition to the theme of poverty for the target population in the study, who at times, could not afford to participate or engage as they would have liked. Interestingly, one publication reports the use of “sanitation fines”, which resulted in a strain on local relationships (Madon et al., 2018). Other factors that contributed to ineffective engagement include food insecurity, lack of ownership of a mobile phone or coverage issues and lack of participant time. This was linked to competing events such as agricultural activities, or local holidays, which in turn are intertwined with the seasons and climate.

### 6.7 Results recorded by study

Both a change in practice or behaviour and an impact on health was reported on by 46.7% of publications. A change in practice or behaviour only was observed in 43.3% of publications and an impact on health alone was seen in 10% of publications.

## 7 Discussion

This scoping review is the first to provide a broad overview of recent CE in remote and rural areas in East Africa. From all the data gathered, a large degree of heterogeneity was observed in the methods used to engage with rural communities, across a wide range of health topics in nine East African countries. Methods of communication varied from a personalized one-on-one interaction to an impersonal mass media approach. A variety of creative methods were used to both mobilize and engage, from cooking demonstrations to songs, and from football games to T-shirts with messages on them. Indeed, most studies used several different methods of engagement rather than just a single intervention in isolation. The range of 30 publications highlighted multiple significant contributory factors that both facilitated and hindered CE, which pertain to the individuals in the community, the environment and the study conditions.

This scoping review serves as a useful resource for anyone wishing to plan an intervention on CE in rural East Africa, particularly for those working on NTDs, which are over-represented in agricultural and unindustrialized locations. This timely publication serves as a buttress for the new road map *Ending the neglect to attain the Sustainable Development Goals: a roadmap for neglected tropical diseases 2021–2030*, endorsed by the Seventy-third World Health Assembly in 2020, which strives to achieve a 90% reduction in people requiring interventions against NTDs and a 75% reduction in disability-adjusted life years related to NTDs by 2030 (WHO, 2020). One of the three pillars of the road map to support these efforts is the scaling up of cross-cutting approaches (WHO, 2020). Furthermore, De Wager et al. (2018) highlight the importance of “quick and tangible wins” to the success of a CE intervention. This scoping review highlights opportunities for quick wins through cross-sectoral collaboration in East Africa; water, sanitation and hygiene (WASH), nutrition, and maternal and child health were common topics of study. WASH has a clear overlap with many NTDs, predominantly in reducing and preventing trachoma, schistosomiasis, lymphatic filariasis, soil-transmitted helminthiases and dracunculiasis (Johnston et al., 2015). This is less obvious for SBE. Nevertheless, WASH plays an important role in good wound care and, where water is scarce, attempts to access a water source could lead to SBE; walking long distances to a water source, often without footwear, increases the chance of inadvertently stepping on a snake. Certain practises during menstruation, such as women being exiled outside of the household when menstruating, have been linked to a risk of SBE (Amatya et al., 2018). Children are also an important demographic to target in combatting SBE; activities such as playing or walking to school place them at increased risk of SBE, and they often suffer more severe envenomations due to their smaller volume of distribution (Le Geyt et al., 2021; Williams et al., 2019). Furthermore, when children are non-verbal it can be particularly difficult to diagnose SBE. Of the 59,000 annual human deaths from rabies, 40% are children living in Asia and Africa (World Health Organization, Food and Agriculture Organization of the United Nations & World Organisation for Animal Health, 2018). Health education in schools should be incorporated into the curriculum, as this is an excellent opportunity to engage with children on the prevention of animal bites, particularly in endemic areas. We can see clear overlay between SBE and current areas where CE is ongoing in rural areas in East Africa. Cross-sectoral collaboration must be encouraged and capitalized on; behaviour change is a lengthy process and project teams need to invest time to develop relationships with communities and build trust. Where a project team has achieved this, they could be supported by another sector for mutual gains that lead to ‘quick wins’. For example, if successful CE on malaria is ongoing, those wishing to engage CE on the prevention of SBE could support the malaria project through provision of bed nets or education materials that also demonstrate how nets keep out snakes that might enter the home. Additional education on other aspects of prevention of SBE could also be included alongside this. This is likely to be more cost-effective than developing a novel CE program in the same area. ‘Quick wins’ should not be confused with an approach that has not be properly considered or is not sustainable. Furthermore, there have been a number of successful interventions designed to prevent and control NTDs including leishmaniasis, leprosy, schistosomiasis, soil-transmitted helminthiases and trachoma, through specifically targeting children (Das et al., 2014; Rujeni et al., 2017). Thus, targeting schoolchildren for CE interventions represents exciting opportunities to educate a group who can act as agents of change on a broad range of health topics for both their families and communities (Milakovich et al., 2018).

This study collected data about who delivered the engagement to the communities; no publications mentioned the involvement of traditional healers, yet the key role that they play in the traditional management of SBE in many communities is well known (Alcoba et al., 2020; Sloan et al., 2007). Although it can be challenging and time-consuming, the NTD community must seek ways to work with traditional healers and community and religious leaders, who hold trusted and influential positions within their communities and thus are key in driving behavioural change (Awah et al., 2018; Kadam et al., 2021). Samuel et al. (2020) demonstrates the efficacy of an education video that includes “snake rescuers” and members of the public who have been bitten by snakes; inclusion of victims of SBE can be a powerful way of engaging with people, and they can act as local champions for the disease. Given the transdisciplinary nature of SBE, veterinarians, herpetologists, snake handlers and snake enthusiasts should also be included in the delivery of CE on SBE, particularly in addressing the impact of SBE on animal health.

This review notes a lack of studies that consider the impact of disease on animal health. The impact of SBE on animal health has traditionally been neglected and this is reflected in the scoping review by Bolon et al. (2019), revealing the underreporting of SBE on animals. Loss of livestock secondary to SBE can have a crippling effect on the livelihood of a rural family, leading to inescapable poverty (Babo Martins et al., 2019). Thus, there is a need to engage with communities on promoting livestock management practices that help prevent snakebite, or indeed other NTDs, in both humans and animals. The WHO NTD road map emphasized the importance of cross-sectoral collaboration, and the transdisciplinary nature of One Health supports this principle (WHO, 2020).

A recent scoping review of how information and communication technologies are used for patient and public involvement in health education in rural areas identified telephone and the Internet as the most commonly used forms (Dogba et al., 2019). Our more comprehensive review found that communication via a mobile phone was used in only 13.3% of studies; use of the Internet was not documented at all. Whilst a number of different methods to engage with people was used in rural East Africa, the most commonly observed method was in a group setting, as a meeting, lecture, presentation or discussion. Whilst Samuel et al. (2020) used Facebook with much success to highlight a campaign on SBE in Tamil Nadu, consideration is needed about the level of mobile Internet connectivity in that geographical area. There remains a significant technological divide, with Sub-Saharan Africa “accounting for almost half of the global population not covered by a mobile broadband network” by the close of 2019, with only 26% of people connected to mobile Internet (Bahia and Delaporte, 2020). As the coverage of mobile networks expands and mobile Internet usage increases, there will be increased scope to harness social media and the Internet for CE on NTDs in rural East Africa.

Communication was identified in this review as the main factor that influenced CE, either positively or negatively. The use of visual aids and culturally-appropriate models were key in communicating with illiterate individuals, and there was difficulty with the explanation of concepts in local languages where certain words, such as cancer, were non-existent, or where explanations concerned internal organs that were hidden (Lidofsky et al., 2019). This is important to consider for SBE, as understanding what happens when a snake bites is key to the explanation of the first-aid management of snakebite. It also underpins a comprehension of why traditional treatments are both ineffective, but potentially harmful. The process of explaining how venom is injected and travels round the body poses a challenge in local languages and should be carefully deliberated to make it culturally relevant. The same is true when explaining that a patient who appears tired may in fact be displaying a bilateral ptosis secondary to a neurotoxic bite.

This study demonstrates the impact of poverty on engagement by affecting rural communities' ability to engage or change their behaviour. Maslow's hierarchy of need clearly outlines that the basic physiological needs, such as food, water and shelter, must be met before other motivations can be considered (Maslow, 1943). Poverty is sadly a recurrent overlapping theme with NTDs; a holistic approach to CE in rural communities is essential to success, taking into account the Sustainable Development Goals and the social determinants of health. This concept is noted in two studies that recommend a package of interventions, targeting more than one area of health in isolation (Ekirapa-Kiracho et al., 2017; Rajasingham et al., 2018).

The involvement of the community in the design of the CE intervention or programme was stressed not only in this review but also in the literature: a set of eight general guiding principles were developed for those seeking to implement a successful community engagement intervention, which stress the early involvement of citizens in a shared decision-making process (De Weger et al., 2018). We observe the significant influence of family and friends on engagement and behaviour change, particularly the influence of grandmothers and husbands on women. Particular attention should be paid to ensuring a CE intervention is inclusive of the whole community and important influencers not only within a community but also within a family or household. This is critical in changing attitudes and opinions around stigma, which are key barriers to engagement.

This study notes that 10% of publications specifically target farmers, an occupation strongly associated with SBE in the tropics. The weather, climate and agricultural activities have negatively impacted attendance at CE interventions in this review; these are barriers of considerable concern, given that those most at risk of SBE are traditionally involved in agrarian activities. CE on SBE should, where possible, avoid the planting and harvesting seasons, as they are associated with a higher burden of work for farmers. Local holidays and festivals also feature as barriers to attendance at events and should be taken into account when planning activities.

Lastly, financial limitations of studies have significantly impacted on their ability to engage communities. Budgeting and financial planning are fundamental components of any intervention and should not be neglected.

The strength of this novel scoping review is derived from its comprehensive and inclusive nature of both human and animal health in rural areas, with no limit placed on the language of publication. By excluding publications that demonstrate only a change in knowledge, attitude or perception, this study eliminates CE that does not necessarily translate to change in behaviour or an impact on health. Whilst this study is limited to East Africa, the results for other geographical areas will be completed as part of the overarching project. The short timeframe of 3.5 years could have been extended; however, given the considerable size of literature on CE the decision was made to limit this to identify more recent, and therefore relevant, CE. It should be noted that there is a probable bias in this review; the approach of the scoping review captures published literature and therefore may not capture effective CE work conducted on a local level by committed and motivated individuals within communities. A limitation of this review is that publications that targeted community health workers were not included, because they were considered as a professional role. However, we note that in some communities these roles are filled by volunteers who work in the community in which they reside; this was often not clear from publications. Where publications demonstrated a non-significant outcome, they were not excluded. It was felt that these studies could also outline relevant contributing factors about the CE that had led to the failure to achieve the intended outcomes. There are many lessons to be learnt from the process of engagement, both positive and negative, regardless of the outcome.

## 8 Conclusion

This study provides an extensive overview of recent public health CE in East Africa that will serve as a useful resource for any group seeking to plan an intervention in remote and rural areas in East Africa. We must consider the elements of good practice in CE more broadly, yet also tailor it to be snakebite-specific. The success of a community education programme is underpinned by an understanding of more than just the disease in isolation; a holistic and comprehensive knowledge of the country and its healthcare system is essential, in addition to the health beliefs, traditions, religions, languages and cultures present. Ultimately, this scoping review will support the development of a WHO CE toolkit for snakebite envenoming and other NTDs. As we concentrate on the impending deadline of 2030, efforts to halve death and disability from SBE must be redoubled, particularly in the wake of the COVID-19 pandemic; CE is a critical component of the multipronged strategy to enable potential victims of SBE to escape death and destitution and to support the recovery of those who have already suffered physical and psychological harm.

**Summary/Recommendations**

• This study provides an overview of current practice of CE on health in rural East Africa.
• Those seeking to undertake CE work on SBE in rural East Africa should consider:
  • a multi-pronged approach using a variety of creative engagement methods to target different sociodemographic groups;
  • a participatory approach to CE;
  • opportunities for ‘quick wins’ through cross-sectoral collaboration;
  • a One Health approach, considering the impact of SBE on livestock and companion animals;
  • local languages, health beliefs, literacy levels, mobile phone ownership and the level of mobile Internet coverage;
  • the most appropriate sociocultural location within the community for CE on SBE; and
  • the impact of climate and agricultural seasons on the ability of farmers to engage.

## Declaration of competing interest

The authors declare that they have no known competing financial interests or personal relationships that could have appeared to influence the work reported in this paper.

## Credit statement

Dr Bethany Moos: Conceptualization, Methodology, Software, Validation, Investigation, Data curation, Writing – Original Draft, Writing – Review & Editing, Visualization, Project Administration, Dr David Williams: Conceptualization, Methodology, Writing – Review & Editing, Visualization, Supervision, Funding Acquisition, Dr Isabelle Bolon: Methodology, Software, Validation, Data Curation, Writing – Review & Editing, Visualization, Dr Denise Mupfasoni: Writing – Review & Editing, Dr Bernadette Abela-Ridder: Conceptualization, Methodology, Writing – Review & Editing, Visualization, Supervision, Funding Acquisition, Dr Rafael Ruiz de Castaneda: Conceptualization, Methodology, Software, Validation, Data Curation, Writing – Review & Editing, Visualization, Supervision, Project Administration.
