# Supplementary material for: A scoping review of current practices on community engagement in rural East Africa: Recommendations for snakebite envenoming
Source: Toxicon X. 2021 Jul 16;11:100073. doi: 10.1016/j.toxcx.2021.100073 (PMC8334718; doi:10.1016/j.toxcx.2021.100073)
Supplement: Multimedia component 1 [file mmc1.docx]

Supplementary material

Appendices

Appendix A. Preferred Reporting Items for Systematic reviews and Meta-Analyses extension for Scoping Reviews (PRISMA-ScR) Checklist

**Preferred Reporting Items for Systematic reviews and Meta-Analyses extension for Scoping Reviews (PRISMA-ScR) Checklist**

| **SECTION** | **ITEM** | **PRISMA-ScR CHECKLIST ITEM** | **REPORTED ON PAGE #** |
| --- | --- | --- | --- |
| **TITLE** | | | |
| Title | 1 | Identify the report as a scoping review. | 1 |
| **ABSTRACT** | | | |
| Structured summary | 2 | Provide a structured summary that includes (as applicable): background, objectives, eligibility criteria, sources of evidence, charting methods, results, and conclusions that relate to the review questions and objectives. | 2 |
| **INTRODUCTION** | | | |
| Rationale | 3 | Describe the rationale for the review in the context of what is already known. Explain why the review questions/objectives lend themselves to a scoping review approach. | 6 |
| Objectives | 4 | Provide an explicit statement of the questions and objectives being addressed with reference to their key elements (e.g., population or participants, concepts, and context) or other relevant key elements used to conceptualize the review questions and/or objectives. | 6 |
| **METHODS** | | | |
| Protocol and registration | 5 | Indicate whether a review protocol exists; state if and where it can be accessed (e.g., a Web address); and if available, provide registration information, including the registration number. | NA |
| Eligibility criteria | 6 | Specify characteristics of the sources of evidence used as eligibility criteria (e.g., years considered, language, and publication status), and provide a rationale. | 7 - 8 |
| Information sources* | 7 | Describe all information sources in the search (e.g., databases with dates of coverage and contact with authors to identify additional sources), as well as the date the most recent search was executed. | 6 |
| Search | 8 | Present the full electronic search strategy for at least 1 database, including any limits used, such that it could be repeated. | 6 - 7 |
| Selection of sources of evidence† | 9 | State the process for selecting sources of evidence (i.e., screening and eligibility) included in the scoping review. | 6 |
| Data charting process‡ | 10 | Describe the methods of charting data from the included sources of evidence (e.g., calibrated forms or forms that have been tested by the team before their use, and whether data charting was done independently or in duplicate) and any processes for obtaining and confirming data from investigators. | 9 |
| Data items | 11 | List and define all variables for which data were sought and any assumptions and simplifications made. | 7, 11 |
| Critical appraisal of individual sources of evidence§ | 12 | If done, provide a rationale for conducting a critical appraisal of included sources of evidence; describe the methods used and how this information was used in any data synthesis (if appropriate). | NA |
| Synthesis of results | 13 | Describe the methods of handling and summarizing the data that were charted. | 9 |
| **RESULTS** | | | |
| Selection of sources of evidence | 14 | Give numbers of sources of evidence screened, assessed for eligibility, and included in the review, with reasons for exclusions at each stage, ideally using a flow diagram. | 7 |
| Characteristics of sources of evidence | 15 | For each source of evidence, present characteristics for which data were charted and provide the citations. | 10 |
| Critical appraisal within sources of evidence | 16 | If done, present data on critical appraisal of included sources of evidence (see item 12). | NA |
| Results of individual sources of evidence | 17 | For each included source of evidence, present the relevant data that were charted that relate to the review questions and objectives. | 10 - 15 |
| Synthesis of results | 18 | Summarize and/or present the charting results as they relate to the review questions and objectives. | 10 - 15 |
| **DISCUSSION** | | | |
| Summary of evidence | 19 | Summarize the main results (including an overview of concepts, themes, and types of evidence available), link to the review questions and objectives, and consider the relevance to key groups. | 15 - 19 |
| Limitations | 20 | Discuss the limitations of the scoping review process. | 19 |
| Conclusions | 21 | Provide a general interpretation of the results with respect to the review questions and objectives, as well as potential implications and/or next steps. | 20 |
| **FUNDING** | | | |
| Funding | 22 | Describe sources of funding for the included sources of evidence, as well as sources of funding for the scoping review. Describe the role of the funders of the scoping review. | 3 |

JBI = Joanna Briggs Institute; PRISMA-ScR = Preferred Reporting Items for Systematic reviews and Meta-Analyses extension for Scoping Reviews.

* Where *sources of evidence* (see second footnote) are compiled from, such as bibliographic databases, social media platforms, and Web sites.

† A more inclusive/heterogeneous term used to account for the different types of evidence or data sources (e.g., quantitative and/or qualitative research, expert opinion, and policy documents) that may be eligible in a scoping review as opposed to only studies. This is not to be confused with *information sources* (see first footnote).

‡ The frameworks by Arksey and O’Malley (6) and Levac and colleagues (7) and the JBI guidance (4, 5) refer to the process of data extraction in a scoping review as data charting*.*

§ The process of systematically examining research evidence to assess its validity, results, and relevance before using it to inform a decision. This term is used for items 12 and 19 instead of "risk of bias" (which is more applicable to systematic reviews of interventions) to include and acknowledge the various sources of evidence that may be used in a scoping review (e.g., quantitative and/or qualitative research, expert opinion, and policy document).

*From:* Tricco AC, Lillie E, Zarin W, O'Brien KK, Colquhoun H, Levac D, et al. PRISMA Extension for Scoping Reviews (PRISMAScR): Checklist and Explanation. Ann Intern Med. 2018;169:467–473. [doi: 10.7326/M18-0850](http://annals.org/aim/fullarticle/2700389/prisma-extension-scoping-reviews-prisma-scr-checklist-explanation).

Appendix B. Full electronic search strategies

Table B.1. Full electronic search strategy for *PubMed*

| Database | Full search string used |
| --- | --- |
| *PubMed* | Title/Abstract  (Rural OR Communit* OR Agricultural OR Agrarian OR Farming OR Farmer) AND (disease OR health OR NTD OR NZD OR Zoono* OR “Snake bite” OR Snakebite) AND (Knowledge* OR Attitude* OR Behaviour* OR Behavior* OR Practice* OR Awareness OR Morbidity OR Mortality OR Death OR Fatality) AND  Title  (Communication* OR “Capacity building” OR Educat* OR Campaign OR Program* OR Toolkit OR Toolbox) |

Table B.2. Full electronic search strategy for *Web of Science*

| Database | Full search string used |
| --- | --- |
| *Web of Science* | TI=((Rural OR Communit* OR Agricultural OR Agrarian OR Farming OR Farmer) AND (Communication* OR “Capacity building” OR Educat* OR Campaign OR Program* OR Toolkit OR Toolbox)) AND  AB=((Disease OR Health OR “Neglected Tropical Disease*” OR NTD* OR “Neglected Zoonotic Disease*” OR Zoono* OR NZD* OR “Snake bite” OR Snakebite) AND (Knowledge* OR Attitude* OR Behavio$r* OR Practice* OR Awareness OR Morbidity OR Mortality OR Death OR Fatality)) |

Table B.3. Full electronic search strategy for *PsycINFO*

| Database | Full search string used |
| --- | --- |
| *PsycINFO* | Title  ((Rural OR Communit* OR Agricultural OR Agrarian OR Farming OR Farmer) AND (Communication* OR “Capacity building” OR Educat* OR Campaign OR Program* OR Toolkit OR Toolbox)) AND  Abstract  ((Disease OR Health OR “Neglected Tropical Disease*” OR NTD* OR “Neglected Zoonotic Disease*” OR Zoono* OR NZD* OR “Snake bite” OR Snakebite) AND (Knowledge* OR Attitude* OR Behaviour* OR Behavior* OR Practice* OR Awareness OR Morbidity OR Mortality OR Death OR Fatality)) |

Table B.4. Full electronic search strategy for *Goggle Scholar*

| Database | Full search string used |
| --- | --- |
| *Google Scholar* | (Rural OR Community OR Agricultural OR Agrarian OR Farming OR Farmer) (Disease OR Health OR NTD OR Zoonosis OR Zoonoses OR NZD OR “Snake bite” OR Snakebite) (Communication OR “Capacity building” OR Education OR Campaign OR Program OR Toolkit) |

Appendix C. Factors that affected engagement

Table C.1. Factors that facilitated engagement

| Factors that facilitated or led to effective engagement | |
| --- | --- |
| Environmental factors | - |
| Financial factors | Income-generating activities have helped to motivate and empower community health workers with resources |
|  | Subsidised price of product |
|  | Use of local inexpensive materials |
| Technological factors | - |
| Communication | The use of behaviour change strategies at multiple levels, including individual, community institutions and mass media |
|  | Using literate individuals to engage with those who were illiterate |
|  | Use of pictures rather than words to engage with those who were illiterate |
|  | Practical use of recipes facilitated engagement with women who were semi‐literate |
|  | Use of visual aids and models to aid understanding |
|  | Culturally appropriate interpretation/translation to preserve cultural context |
|  | Sensitisation/mobilisation meetings prior to intervention |
|  | Involvement of important influencers |
|  | Social marketing of product |
|  | Free monthly calendars containing information about different health topics |
| The intervention | A package of interventions, rather than a single intervention |
|  | A combined strategy of nutritional education and agricultural support, skills and technology, rather than targeting one area in isolation |
|  | A participatory approach |
|  | Use of a participatory approach |
|  | Community participation in decision-making and program implementation |
|  | Demonstration of the ease of use and taste of the new product is the same |
|  | Rewarding users by offering incentives, can improve outcomes (even when incentives were out of stock) |
| Location of intervention | Health clinic as a choice of location, as it is regarded as a trusted source of information |
| People delivering the intervention | Personal characteristics of those delivering the intervention, particularly enthusiasm and confidence |
|  | Seeing the trainers being supervised encouraged participants to take intervention seriously |
|  | Appropriate and adequate support for community nutrition promotors to perform their role well |
|  | Study team comprised 50% of local Tanzanians to increase acceptability |
|  | Flexibility for the those delivering the intervention, which allows a sense of ownership |
| Delivery of intervention | Clear self-collection instructions (of cervical specimen) and adequate privacy to do so |
|  | Delivery of education through existing community groups |
|  | Demonstrations of handwashing procedure |
| Cultural factors | Models dressed in traditional clothing |
| The wider community | The influence of family and friends and the influence of the person delivering the message (can impact either positively or negatively) |
|  | Social support to help practises be adopted and maintained |
|  | More regular interactions between the social services committee and the village general assembly, health facilities, livestock and water experts also suggest a greater sense of empowerment as the social services committee is perceived as a credible village body |
| Other | The desire from caregivers to improve the health status of their children |
|  | Children liked the taste of product |
|  | Awareness of services and their quality |
|  | Medical treatment facilitated compliance with recommendations |

Table C.2 Factors that hindered effective engagement

| Factors that hindered or led to ineffective engagement | |
| --- | --- |
| Environmental factors | Weather/climate |
|  | Weather/climate |
|  | Weather/climate and agricultural activities |
|  | Increased parent–child conflict due to demands on children to assist with farming activities or caregiving of younger children |
|  | Reduced parent engagement and responsiveness due to increased agricultural activity |
| Financial factors | Poverty |
|  | Financial limitations of study |
|  | Study budgetary constraints |
|  | Cost of product |
|  | The imposition of sanitation fines |
|  | Financial constraints impacted access to treatment |
|  | Cost of food in improving dietary intake and nutritional status |
|  | Perceived corruption due to lack of transparency and accountability |
| Technological factors | Mobile network coverage issues |
|  | Lack of ownership of a mobile phone |
| Communication | Language of written materials |
|  | Inadequate communication/instructions delivered about intervention |
|  | Difficulty explaining the concept of organs, because they are hidden inside the body and unseen |
|  | Different local languages required a trilingual interpreter |
|  | Lack of written form of local language |
|  | Lack of equivalent words in local language eg. cancer |
|  | Short duration of community mobilization conducted prior to education |
|  | A long period of time is required to achieve sustainable behavioural change |
| The intervention | Poor quality of resources |
|  | Quantity of supplements used in study was too small |
| Location of intervention | - |
| People delivering the intervention | Those delivering intervention are volunteering their time, rather than being paid |
|  | Financial incentives for CHWs not large enough to significantly increase their income |
| Delivery of intervention | Distance to the health facility |
|  | Lack of means of transport impacts access to treatment |
|  | Competing activities in the community |
| Cultural factors | Local beliefs affected behaviour change |
|  | Local beliefs and practices |
|  | Stigma impacts access to treatment |
|  | Stigma is associated with being a victim of violence |
|  | Particular syndrome is associated with social stigma |
|  | Holiday season - fewer events held and a lower volume of product redeemed |
| The wider community | Unsupportive community |
|  | Hierarchy/authority within families |
|  | Husbands determined their wife's participation in education sessions |
| Other | Scarcity of food |
|  | Food shortage |
|  | Food insecurity in study area |
|  | Resource constraints and limited food diversification |
|  | Lack of time |
|  | Women's workload and time constraints |
